# Examining the Relationship Between Leaders' Power Use, Followers' Motivational Outlooks, and Followers' Work Intentions

**DOI:** 10.3389/fpsyg.2018.02620

**Published:** 2019-02-01

**Authors:** Taylor Peyton, Drea Zigarmi, Susan N. Fowler

**Affiliations:** ^1^School of Hospitality Administration, Boston University, Boston, MA, United States; ^2^Valencore Consulting, Cambridge, MA, United States; ^3^The Ken Blanchard Companies, Escondido, CA, United States; ^4^University of San Diego, San Diego, CA, United States

**Keywords:** power, motivation, self-determination theory, work intentions, leader power, soft power, hard power

## Abstract

From the foundation of self-determination theory and existing literature on forms of power, we empirically explored relationships between followers' perceptions of their leader's use of various forms of power, followers' self-reported motivational outlooks, and followers' favorable work intentions. Using survey data collected from two studies of working professionals, we apply path analysis and hierarchical multiple regression to analyze variance among constructs of interest. We found that followers' perceptions of hard power use by their leaders (i.e., reward, coercive, and legitimate power) was often related to higher levels of sub-optimal motivation in followers (i.e., amotivation, external regulation, and introjected regulation). However, followers who perceived their leaders used soft power (i.e., expert, referent, and informational power) often experienced higher levels of optimal motivation (i.e., identified regulation and intrinsic motivation), but further investigation of soft power use is warranted. The quality of followers' motivational outlooks was also related to intentions to perform favorably for their organizations.

## Introduction

This study merges two fields of investigation: forms of leadership power stemming from empirical research on the psychology of power over the last five decades, and motivational outlooks from research on self-determination theory (SDT) over the last 40 years. Researchers in both areas have called for greater in-depth exploration of the relationship between leadership and motivation in organizational settings (eg., Elias, 2008; Stone et al., 2009; Meyer et al., 2010; Randolph and Kemery, 2011; Anderson and Brion, 2014).

Much of the research on the psychology of power is “still largely removed from the complexities and confounds of behavior in organizational settings” (Anderson and Brion, 2014, p. 85). While laboratory studies have certain strong advantages, future research will need to grapple with the dynamics of interpersonal and psychological interactions, and related implications for organizational life (Anderson and Brion, 2014).

Podsakoff and Schriesheim (1985) called for investigations of the independent contributions different power bases make to explain variance in criterion variables relevant to subordinate outcomes. Also, Elias (2008) identified a need for more research on specific criteria that facilitate leaders' decisions regarding the kind of power they should exercise. In response to these calls and other apparent gaps in the literature, studies began to appear. For example, Mossholder et al. (1998) found that subordinates' perceptions of procedural justice fully mediated the relationship between ratings of their supervisors' use of five forms of social power, and job satisfaction and organizational commitment. Ward (1998) also studied subordinates' perceptions of their managers, and found that for four of eight aspects of psychological work climate, managerial power bases interacted with subordinates' manifest needs (achievement, dominance, autonomy, and affiliation). Notably, managers' use of personal power (expert and referent) had the biggest impact on psychological climate, especially when personal power use also occurred with reward power use (Ward, 1998). Politis (2005) examined relationships between five forms of managerial power and credibility, with employee knowledge acquisition attributes. Politis (2005) uncovered a positive relationship between expert power and knowledge acquisition attributes including negotiation, control, and personal traits; also, the study found that greater use of coercive and referent power related to lower levels of knowledge sharing and knowledge acquisition. Additionally, Pierro et al. (2012) discovered how supervisors' and subordinates' need for cognitive closure related to the efficacy and application of various social power bases, specifically regarding: employee preference for soft or hard power, subordinates' performance, and other organizational outcomes. Pierro et al. (2013) found positive relationships between transformational/charismatic leadership and subordinates' inclinations to comply with soft power, which was also indicative of higher levels of affective organizational commitment.

While the above studies demonstrate some of the work on power bases as they relate to criterion variables in accordance with calls from Podsakoff and Schriesheim (1985) and others, very few studies examine the connection between motivation and forms of power use, and SDT has not yet been comprehensively applied in these investigations. Power is an undeniable aspect of leadership, and we agree with other authors (i.e., Aguinis et al., 1994; Randolph and Kemery, 2011) who maintain that there is not enough is known about the degree to which employee perceptions of their managers' use of various forms of power is correlated with various forms of employee motivation.

Scholars in the field of SDT have made similar calls for more in-depth research connecting leadership behavior to motivation in organizational settings (e.g., Deci and Ryan, 2002; Stone et al., 2009). Some SDT researchers have requested a closer examination of how leadership qualities and interpersonal styles of managers influence their followers' tendencies to align their personal goals with organizational goals (i.e., Gagné and Deci, 2005). Other SDT researchers call for the examination of how leader behaviors can foster increased levels of intrinsic motivation in followers (e.g., Jungert et al., 2013). Still other SDT authors ask for research on how leaders might optimize employee engagement in organizational settings (Dysvik and Kuvaas, 2010).

A preponderance of the SDT literature and its core principles has been applied in settings other than business (Deci and Ryan, 2002; Ryan and Deci, 2017). While some business leaders may have a cursory awareness of the fundamental concepts of SDT (such as the basic psychological needs of autonomy, relatedness, and competence), few understand how to successfully support employees' needs in the face of organizational pressures for performance and output (Stone et al., 2009). Much of the present organizational psychology depends upon the “carrot and the stick” strategy for motivation and command-and-control methods for performance and behavior (Stone et al., 2009; Fowler, 2014). Here we explore the impact of various kinds of leadership power on non-power holders in organizations. We are interested in gaining insight into how a non-power holder's motivational outlooks may relate to their leaders' use of power in the workplace.

### Purpose and Organization of the Study

This study aims to answer calls from researchers in both fields of investigation by empirically examining the relationship between leaders' use of various forms of power, followers' motivational outlooks, and followers' work intentions in organizations. Specifically, we propose to answer Podsakoff and Schriesheim (1985) call “to assess the independent contribution of each of the power bases to the variance explained in subordinate criterion variables” (p. 406). This study, is also directed toward the third research avenue suggested by “to consider putting future research efforts into those who are powerless” (Strum and Antonakis, 2015, p. 157). In response, we focus on the intrapersonal experiences of followers who operate under their leaders' power. We examine the degree to which the perceived use of six forms of leader power might explain variance in follower motivation (i.e., its sub-forms), and follower work intentions. We consider existing literature on power and SDT to hypothesize that leaders' use of different kinds and combinations of power is connected to various motivational outlooks and work intentions in the non-power holder.

## Review Of The Literature On Leader Power, Employee Motivation, And Employee Work Intention

### Leader Power

Power typically entails a condition in which some individuals have control over resources and some do not. The term power is most commonly defined as “the asymmetric control over valued resources” (Anderson and Brion, 2014, p. 69). Power relationships are inherently social and exist only in relation to others; parties with low power rely on parties with high power to obtain rewards and avoid punishment (Vince, 2014).

For leaders to be effective they must be able to shape the behavior of others (Elias, 2008). Forms of leader power that can be used to shape others behavior are embedded in people's psyches (Vince, 2014) through the structural features of today's organizations (Pfeffer, 1992; Clegg et al., 2006; Vince, 2014). Before the turn of this century, much of the literature concerned with leader power has been sociological or philosophical in origin and character (Elias, 2008; Anderson and Brion, 2014) and has traditionally dealt with the “social structure of corporations” (Clegg et al., 2006, p. 387). The structural perspective of power (Pfeffer, 1992) has given rise to the foundation of systems of authority and the formation of legitimate social power relationships (e.g., Pfeffer, 1992, 2011; DuBrin, 2009; Haugaard and Clegg, 2012; Lunenberg, 2012; Vince, 2014).

Leaders in the workplace may reinforce their power through their own demeanor and behavior, but they are ordained with their power by the organizational context (e.g., the authority to control resources, followers assigned to work under them), which includes higher-ranking small groups or strategic decision makers (Anderson and Brion, 2014). The antecedents for the bases for power stem from the social structure, the cultural patterned behavior of groups, and other practices within organizations (Lukes, 1974, 2005). Much of the early literature on power was concerned with the character, skills and personality of the designated leader, with the organization's structures, policies, procedures, and with forms of hierarchy that authorize a leader's power (Foucault, 1982).

The bases of power (French and Raven's, 1959; Raven, 1965), which will be described next, define forms of power observed from human and contextual factors, whereas the psychology of power is concerned with how people perceive and experience the power bases, either when they hold the power themselves, or when they are under the power of others. Since the beginning of the twenty-first century, there has been a rise in the number of empirical studies on the psychology of power, as researchers have recently begun to explore psychological perceptions of power to better understand how power use affects individuals in an organizational context (Anderson and Brion, 2014). There has been a shift away from studying power as it resides in structures, policies, and procedures, and a shift toward studying individual perceptions that are held by power/non-power holders when various uses of power occur. Recent work on individual perceptions includes, for example: studies concerned with why power facilitates self-interested behavior (DeCelles et al., 2012); how people who are primed with high- vs. low-power tend to adopt the visual perspective of others, adjust to other people's points of view, and feel empathy for others (Galinsky et al., 2006); and how power influences people's thinking while resolving moral dilemmas (Lammers and Stapel, 2009). Additionally, we agree with other researchers (e.g., Farmer and Aguinis, 2005; Dambe and Moorad, 2008) who have noted that most studies on power have predominantly focused on the power holder and not on either the mutuality of the power holder and the non-power holder, or on the perspective of the individual non-power holder.

#### Various Bases of Power

French and Raven's (1959) and Raven (1965) presented five conceptual forms of leader power which have been the basis of 50 years of research (Elias, 2008). These five forms of leader power—expert, referent, reward, legitimate, and coercive power—have remained relatively constant over time, even though there have been controversial issues, such as response bias possibilities, concept overlap, and single-item measurement (cf. Podsakoff and Schriesheim, 1985). To date, French and Raven's (1959) five forms of power are frequently used for the study of power in organizations.

Subsequently, Raven refined the five concepts of power from the earlier 1959 publication by adding a sixth major form of power, informational power, and further differentiated types of legitimate power into legitimate reciprocity, legitimate equity, and legitimate dependence (Raven, 1993). Additionally, coercive power was divided into personal coercive power and impersonal coercive power (Raven et al., 1998), and reward power was separated into personal reward power and impersonal reward power (Raven et al., 1998). These changes were made in an effort to overcome some of the concerns raised by Podsakoff and Schriesheim (1985) and Yukl and Tracy (1992), among others (Raven et al., 1998).

Alternatively, some researchers have classified varying forms of power into two clusters, i.e., soft and hard power, based on the amount of perceived freedom employees have in responding to the types of power used by their managers (e.g., Raven et al., 1998; Pierro et al., 2008; Randolph and Kemery, 2011). Expert power, informational power, and referent power are referred to by these and other authors as soft forms of power, while coercive, reward, and legitimate power have been classified as hard forms of power. Hard types of power require higher levels of non-power holder compliance and result in lower levels of autonomy.

#### Soft Power

Expert power depends on perceptions of the follower regarding the influencer's superior knowledge (Raven et al., 1998). The strength of this power turns upon the amount of expertise or knowledge the follower attributes to the influencer on a specific topic (Podsakoff and Schriesheim, 1985). Informational power refers to the influencer's perceived capacity to provide a rationale to the follower regarding why the follower should change his or her beliefs or behaviors (Raven et al., 1998). Referent power is dependent on the follower's perceived personal identification with the influencer (Raven et al., 1998). The basis of this power stems from the extent to which the follower's personal self-identity is made better through interaction with the influencer or the desire of the follower to be like, or associated with, the influencer (Podsakoff and Schriesheim, 1985).

#### Hard Power

Coercive power is defined as the perceived ability of the leader to penalize the targets if they do not adhere to requested outcomes (Raven et al., 1998). The potency of coercive power lies in the perceived extent of the punishment possible, and its use often correlates with increased negative affect between leader and follower (Podsakoff and Schriesheim, 1985). Reward power originates from a perceived possibility of monetary or non-monetary compensation (Raven et al., 1998). The intensity of reward power heightens with an increase in the rewards possible, and with its relative attractiveness to the receiver (Podsakoff and Schriesheim, 1985). Legitimate power originates from the subordinate's perceived understanding of the leader's right to influence (Raven et al., 1998). The potency of legitimate power arises from the internalized values the follower has concerning the authority or right of the leader to be the leader (Podsakoff and Schriesheim, 1985).

#### Correlates and Outcomes of Power

The nature of power is inherently a double-edged sword in which some people have prerogatives and others do not. This realization has given rise to research on self-interested behavior and the misuse of various forms of power, the subsequent examination of psychological explanations (e.g., Galinsky et al., 2006; Lammers and Stapel, 2009; DeCelles et al., 2012), and resultant possible solutions, such as empowerment (e.g., Conger and Kanungo, 1988; Randolph and Kemery, 2011).

Research studies in the last two decades reveal that the use of various forms of power correlates with various desirable and undesirable organizational and individual outcomes. For example, greater use of soft kinds of power (expert, referent, and informational power) are connected to higher levels of organizational citizenship behavior, empowerment, organizational commitment, and job satisfaction (e.g., Podsakoff and Schriesheim, 1985; Elias, 2008; Randolph and Kemery, 2011), whereas the use of hard forms of power (coercive, reward, and legitimate) are related to greater absenteeism, lower productivity, lower self-confidence levels, and burnout (Podsakoff and Schriesheim, 1985; Elias, 2008; Randolph and Kemery, 2011).

### Employee Motivation and Self-Determination Theory

The concept of motivation in this study originates from the fundamental tenets of SDT, which have been researched and confirmed in the past five decades. This theory holds that individuals are volitional, able to initiate behaviors (Deci and Ryan, 1985, 2002) and that individuals thrive when their psychological needs are satisfied (Deci and Ryan, 1985, 2002). SDT purports that the individual cognitively process their experience which results in self-direction through flexible psychological structures that allow individuals to direct action toward the achievement of desired ends (Ryan and Deci, 2017).

Additionally, SDT researchers (e.g., Deci and Ryan, 1985; Ryan and Deci, 2000) maintain that an individual's actions are self-determined when they are chosen and supported by personally defined boundaries rather than being coerced, pressured, or induced through incentives. The term self-determination connotes a sense of self-management or self-regulation that, over time, brings with it goal direction, energy, persistence, and intention (Ryan and Deci, 2000, 2017). In other words, individuals can understand why they behave the way they do. SDT purports that individuals can understand the causality of their actions, develop causality orientations (implicit and explicit, Deci and Ryan, 1985), and regulate their future behaviors to be congruent with such orientations.

SDT emphasizes that individuals' psychological needs for autonomy, relatedness, and competence must be fulfilled (Deci and Ryan, 1985, 2002). Rather than focusing upon the lessening of physiological drives of sex, hunger, thirst, and pain avoidance, lasting human motivation originates from intrinsic needs for integration and growth (Deci and Ryan, 1985). Given that there is a spectrum of needs, frequent interactions with the environment allow for the fulfillment of basic psychological needs for thriving and flourishing, that encompasses more than physiological satiation (Deci and Ryan, 2002). The basic psychological needs of autonomy, relatedness, and competence, often designated as “psychological nutriments,” are as mandatory as physiological nourishment for human psychological development and well-being (Ryan and Deci, 2000, p. 75). These basic psychological needs can give rise to various forms of motivational regulation and their associated motivational outlooks.

SDT defines two broad categories of motivational regulation: controlled regulation and autonomous regulation. Controlled regulation entails participation in an activity for instrumental reasons, rather than for reasons of pleasure or being interested in the activity for the sake of the activity itself (Gagné and Deci, 2005; Meyer et al., 2010). Autonomous regulation is designated as a person's participation in an activity for its own sake, because it is pleasurable or because it is of interest (Gagné and Deci, 2005; Meyer et al., 2010). Within controlled and autonomous regulation, SDT postulates various sub-categories or motivational outlooks: external, introjected, identified, and intrinsic. In this paper, we use the terms for motivation, such as motivational outlooks or forms regulation offered by Gagné et al. (2015).

An external motivational outlook is driven by desired rewards or punishment avoidance (i.e., controlled regulation). An introjected motivational outlook is connected to ego enhancement or to the avoidance of guilt or shame (i.e., controlled regulation). External and introjected motivational outlooks are classified as controlled regulation, in that they originate from instrumental outcomes or external conditions (Gagné and Deci, 2005).

An identified motivational outlook is a state in which the individual participates in activities to be congruent with valued personal goals (i.e., autonomous regulation). The identified motivational outlook usually stems from willful actions that adhere to stated values. If, after reflection, the individual believes he/she has chosen at will to engage in an activity because it is congruent with his/her fundamental needs and values, a sense of autonomy is obtained (Ryan and Deci, 2000; Gagné and Deci, 2005). And finally, an intrinsic motivational outlook is a state in which a personal sense of self is expressed by the individual when participating in an activity (i.e., autonomous regulation) (Gagné and Deci, 2005; Meyer et al., 2010). Identified or intrinsic motivational outlooks are categorized as autonomous regulation.

Apart from the motivational regulations, a state of amotivation, or disinterest, can occur when people lack the volition to act—or act passively—toward a specific outcome. Amotivation may exist because of forces beyond the individual's control. This feeling of helplessness may stem from uncontrollable or unpredictable environmental factors, or it could happen because the individual was overwhelmed by thoughts and feelings from within, such as anger, rage, resignation, or despair (Deci and Ryan, 1985). It is possible that employees may carry out their tasks mindlessly and without purpose or care, with little regard for their performance. External and introjected regulation are different from amotivation because, in the former, the motivation of the individual expresses a modicum of volition for some specific outcome.

We use the language “sub-optimal” and “optimal” to broadly refer to clusters of motivational outlooks or states, and the distinction is based on each state's support of sustainable, long-term human flourishing. Sub-optimal motivational outlooks include amotivation, external regulation, and introjected regulation; they are classified together as sub-optimal, in that an individual's energy toward a given task is approached from a lack of interest, or from a psychological place other than positive interest or value-congruent reasons. Thus, employee performance originating from any of the sub-optimal motivational outlooks, over the long haul, will either be characterized by a lack of effort or will likely not be sustainable. Alternatively, we classify identified and intrinsic motivational outlooks (i.e., autonomous regulation) as optimal, because they involve greater fulfillment of basic psychological needs, and therefore employee efforts stemming from optimal motivation are more likely to be sustainable.

### Work Intentions

In keeping with the SDT, an individual's energy for “volitional, intentional behavior originates from underlying personal needs for autonomy, relatedness, and competence” (Deci, 1980, p. 23). Here an intention is defined as a mental image of the behavior an individual plans to manifest (Bagozzi, 1992). Several studies have revealed that intentions are an important concept in the attitude-intention-behavior chain (e.g., Ajzen and Fishbein, 1980; Armitage and Connor, 2001). Studies testing social cognitive appraisal theory, for instance, have predicted work satisfaction from self-efficacy, positive affect, and work conditions (Duffy and Lent, 2009), have examined the relationship between control coping and employee withdrawal during organizational change (Fugate et al., 2011), have identified the relationship between appraisal/coping variables and stressful encounter outcomes (Folkman et al., 1986), and have uncovered relationships between consumers' behavioral intentions to use services in the future with consumer expectations, perceived quality, and satisfaction (Gotlieb et al., 1994).

In the fields of health and social psychology, various meta-analyses have demonstrated strong relationships between intentions and behavior (e.g., Cooke and Sheeran, 2004; Gollwitzer and Paschal, 2006; Webb and Sheeran, 2006). We chose to use the concept of work intentions as outcome variables because they are stronger predictors of employee behavior. Three meta-analyses conducted over the last 40 years have established that intentions are better predictors of employee behavior than outcome variables, such as organizational commitment and job satisfaction (e.g., Steel and Ovalle, 1984; Tett and Meyer, 1993; Podsakoff et al., 2007).

## Hypotheses

**Figure 1:**
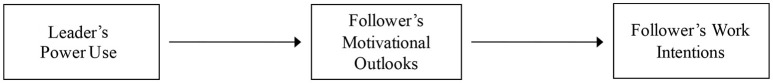
Overall conceptual model.

This research explores possible relationships between followers' perceptions of their leader's use of different kinds and combinations of power, various types of motivational outlooks in followers, and five work intentions held by followers. See Figure 1 for our conceptual model.

Very little empirical testing of the relationships between power and motivation exists in the literature, so the main impetus for our hypotheses is theoretical. Our underlying logic regarding followers' motivational outlooks assumes that the non-power holders' basic psychological need for autonomy, relatedness, and competence will be met or not met, facilitated are not facilitated, through the leader's use of various forms of power, hard or soft. The theoretical justification for these hypotheses lies in the followers' experience of the quality of: choice or autonomy given by the leader, relatedness cultivated by the leader, and competence experienced in relationship to the leader.

Specifically, we point to the research on human choice. Building on the work of SDT researchers, Patall et al. (2008) included 41 studies in a meta-analysis examining the effects of choice on intrinsic motivation. The authors concluded that intrinsic motivation was stronger when choice was given, and when rewards were not given. According to SDT, when a person's basic psychological needs (autonomy, relatedness, competence) are met, they thrive and behave in more self-determined (or optimally motivated) ways. Giving a person a choice relates to their experience of autonomy, one of the three basic psychological needs. Definitions of various forms of power (i.e., Podsakoff and Schriesheim, 1985; Raven et al., 1998; Pierro et al., 2008) suggest that coercive power, reward power, and legitimate (i.e., hard) power provide limited opportunity for the person under these types of power to exercise choice, compared to expert, referent, and informational (i.e., soft) power. If a leader's hard power limits follower choice much more than soft power, we anticipate followers' basic psychological needs and motivation will be affected accordingly.

Also, we found two empirical studies that examined different forms of power and motivation. First, Elangovan and Xie (1999), reported many positive relationships between subordinates' levels of internal motivation and forms of power being used by their supervisors, but effects were notably stronger for subordinates with low self-esteem or external locus of control. Second, Pierro et al. (2008) reported hard power compliance positively correlated with extrinsic motivation and negatively correlated with intrinsic motivation, whereas soft power compliance was positively correlated with intrinsic motivation. Pierro et al. (2008) and Elangovan and Xie (1999) both did not frame their studies primarily in SDT, so neither study included measures of motivation that captured the various subscales of motivation comprehensively defined by SDT.

Given the small number of studies exploring the relationship between leader power and subordinate motivation, more research is needed. Thus, applying conclusions from the research on human choice, from SDT, and from the above literature testing French and Raven's power bases in relationship to motivation, we propose:

*Hypothesis 1a*: Leaders' use of various kinds of hard power will positively correlate with followers' sub-optimal motivation (i.e., amotivation, external regulation, introjected regulation).
*Hypothesis 1b*: Leaders' use of various kinds of hard power will negatively correlate with, or not correlate with, followers' optimal motivation (i.e., identified regulation, intrinsic motivation).
*Hypothesis 1c*: Leaders' use of various kinds of soft power will negatively correlate with, or not correlate with, followers' sub-optimal motivation (i.e., amotivation, external regulation, introjected regulation).
*Hypothesis 1d*: Leaders' use of various kinds of soft power will positively correlate with followers' optimal motivation (i.e., identified regulation, intrinsic motivation).

Furthermore, leaders' use of power and followers' motivation may also relate to followers' work intentions. Zigarmi et al. (2016) examined employee locus of control, forms of motivational regulation, harmonious and obsessive passion, and desirable work intentions. While the direct connection between motivational regulation variables and work intentions in Zigarmi et al. (2016) was not estimated in their structural model, their correlation matrix showed: strong positive relationships between autonomous motivation and all five work intentions, small-to-medium negative relationships between amotivation and all work intentions, and small positive relationships between controlled motivation and three of five work intentions. Such relationships are in accordance with the assumptions of SDT, which purport that optimal motivation relates to human thriving.

Additionally, Zigarmi et al. (2015) found slight-to-moderate positive correlations between employees' perceptions of their leaders' use of expert and referent (i.e., soft) power and all favorable work intentions. In the same study, coercive and legitimate (i.e., hard) power were negatively and somewhat weakly correlated with five of ten possible work intentions, whereas reward power was somewhat positively correlated to all five work intentions. In their structural model that included positive and negative affect as mediators, Zigarmi et al. (2015) found that—except for expert power which had a negative, direct path to intent to perform—reward power and expert power each positively and directly related to two of five favorable work intentions. From that work, we observe variability in employees' intentions to perform well for their organization, relative to the kind of power their leaders use.

Considering the above, regarding the relationship between power, motivation, and work intentions, we propose:

*Hypothesis 2a*: Followers' sub-optimal motivation (i.e., amotivation, external regulation, introjected regulation) will negatively correlate with, or not correlate with, their work intentions.
*Hypothesis 2b*: Followers' optimal motivation (i.e., identified regulation, intrinsic motivation) will positively correlate with their work intentions.
*Hypothesis 3a*: Followers' motivational outlooks will partially mediate perceptions of leaders' use of various kinds of power and followers' work intentions.
*Hypothesis 4a*: Followers of leaders who use multiple kinds of hard power at high levels (as compared to leaders who use lower levels of all kinds of hard power) will report higher levels of amotivation, external regulation, and introjected regulation, lower levels of (or no difference in levels of) identified regulation and intrinsic motivation, and lower levels of (or no difference in levels of) work intentions.
*Hypothesis 4b*: Followers of leaders who use multiple kinds of soft power at high levels (as compared to leaders who use lower levels of all kinds of soft power) will report higher levels of identified regulation and intrinsic motivation, lower levels of (or no difference in levels of) amotivation, external regulation, and introjected regulation, and higher levels of work intentions.

*Hypothesis 3a* was written parsimoniously to address all substantive constructs of interest to us, as our approach to partial mediation analyses will be exploratory. Thus, any non-significant relationships we uncover from testing *Hypotheses 1a*−*1d* and *2a*−*2b* will naturally affect the possibility to test partial mediation proposed by Hypothesis 3a.

In summary, we hypothesize that a leader's increased use of harder forms of power will be related to decreased quality of their followers' forms of motivation, and that less optimal motivation in employees will relate to lower levels of work intentions. Also, the more optimal forms of motivation in employees should correlate with higher levels of work intentions. While various studies we cited above provide some support for the connection between followers' work intentions relative to their motivation, and to their leaders' use of power, we have found no empirical study yet that has examined these factors together.

## Methods

We conducted two studies to test our hypotheses. Study 1 involved a sample of respondents from a single organization, while Study 2 collected a larger sample of employees working across many organizations. Study 2 was conducted to determine if the findings from Study 1 could be replicated. Both studies were approved by the Research Ethics Committee of The Ken Blanchard Companies.

### Participants for Study 1

Three-hundred seventy employees from a training and consulting organization in Southern California were invited to participate in Study 1. The sample for analysis included 229 employees, or a 62% response rate. Seventy percent of respondents were female, 78% were White/Caucasian, 22% were managers, and 60% reported being born in 1961 or later. Thirty percent had graduate degrees, 44% were college graduates, and 26% had some college education or less. Organizational tenure varied; 30% said they had been with their organization for 5 years or less, 21% reported a tenure of 6–10 years, 34% reported a tenure of 11–20 years, and 15% said they had worked for their organization for 21 years or more.

### Procedures for Study 1

Participants from a single organization were invited through email to complete an online survey. Data for this study were gathered as part of a voluntary, anonymous, annual survey conducted by the company's human resources department.

#### Measures

In addition to demographic information, participants were asked to respond to subscales measuring their manager's use of power and the kinds of motivational outlooks they personally experience at work.

#### Power

Managerial use of power was measured through the Interpersonal Power Inventory (IPI) from Raven et al. (1998). The IPI presents 11 subscales representing various power bases and is an extension of the original six power bases proposed by French and Raven's (1959) and subsequently Raven (1965). The IPI asks respondents to think of a time when they complied with their supervisor's request despite initially being reluctant to do so, then presents 33 items asking for their reason for compliance to be rated on a 7-point response scale (1 = *definitely not a reason*, 7 = *definitely a reason*). To ensure parsimony and practicality in the interpretation of results, Study 1 combined the IPI's eleven subscales of power to measure the original six power bases: reward power, coercive power, legitimate power, expert power, referent power, and informational power. The reward and coercive power subscales each had six items, the legitimate power subscale included nine items, and the expert, referent, and informational power subscales were each made up of three items. Example items follow for each kind of power subscale used in this work: “My supervisor could help me receive special benefits” (reward power), “My supervisor may have been cold and distant if I did not do as requested” (coercive power), “I understood that my supervisor really needed my help on this” (legitimate power), “My supervisor probably knew more about the job than I did” (expert power), “I looked up to my supervisor and generally modeled my work accordingly” (referent power), and “My supervisor gave me good reasons for changing how I did the job” (informational power). Alpha coefficients for the power subscales in Study 1 ranged from 0.87 to 0.96.

#### Motivation

Employee workplace motivation was measured using the Multidimensional Work Motivation Scale (MWMS). This 19-item work scale has been validated in seven languages (see Gagné et al., 2015). Participants were asked to respond to the item “why do you or would you put efforts into your current job” and were given a 7-point rating scale (1 = *not at all*, 7 = *completely-entirely*) to indicate the degree to which each survey item represented their reasons for expending the effort to become involved with their job. The MWMS includes six subscales for workplace motivation: amotivation, external-social, external-material, introjected, identified, and intrinsic. For this study, researchers combined the external-social and external-material subscales to create a total score for external regulation, such that we included five dimensions for motivation. Three, six, four, three, and three items, respectively, made up our five forms of motivational outlooks: amotivation, external regulation, introjected regulation, identified regulation, and intrinsic motivation subscales. An example item for motivation is “Because the work I do is interesting” (intrinsic motivation).

For Study 1, alpha coefficients for amotivation and introjected regulation were below 0.70, and respective results should, therefore, be interpreted with caution. Gagné et al. (2015) tested the psychometric properties of all MWMS subscales in various cultural contexts and cited borderline or inadequate reliability (≤0.70) for the introjected regulation subscale in three of seven samples. However, Gagné et al. cited adequate reliability (>0.70) for the amotivation subscale in all samples, so issues with the measurement of amotivation were surprising. The reliabilities of all other motivation subscales for Study 1 were adequate, ranging from 0.80 to 0.90.

Also, in Study 1 there was a strong positive skew of the amotivation subscale, which was initially problematic for analysis; for the three items for this subscale, 81–89% of respondents indicated that they were “not at all” experiencing amotivation at work. To work with the data, we dichotomized the amotivation items so that those who were experiencing no amotivation at all were coded in a separate category from respondents reporting any level of amotivation at work, and subscale average total scores were calculated from these variables prior to analysis. This treatment of the data was beneficial because it lessened the strength of the positive skew of the amotivation variable to be used in subsequent regression analyses.

#### Work Intentions

Employee work intentions data were collected from the short form of the Work Intentions Inventory (WII) (Nimon and Zigarmi, 2015), and this inventory was included within the surveys used for Studies 1 and 2. Over the past 4 years, two versions of the work intentions inventory have been developed. The initial WII long form (Zigarmi et al., 2012) contained 25 items while the WII short form included 15 items. In each WII version, 5 types of work intentions are represented (Nimon and Zigarmi, 2015): intent to use discretionary effort, intent to perform at a higher than average level, intent to endorse, intent to stay with the organization, and intent to use organizational citizenship behavior. The WII has demonstrated construct and content validity for the five work intentions subscales and has repeatedly displayed appropriate internal consistency and factorial structure (Nimon and Zigarmi, 2015).

The WII offers a six-point Likert-type response scale format to capture the respondent's extent of intention experienced, ranging from *1* = *no extent* to 6 = *the fullest extent*. For the WII short form used in this study, each of the five work intentions subscales were measured with three items. Example items include: “I intend to take home work when I know it will make me more effective the next day” (intent to use discretionary effort), “I intend to exert the energy it takes to do my job well” (intent to perform), “I intend to talk positively about this organization to my friends and family” (intent to endorse), “I intend to continue to work here because I believe it is the best decision for me” (intent to stay), and “I intend to watch out for the welfare of others at work” (intent to use OCB). In this work, reliabilities for the five intentions subscales ranged from 0.77 to 0.93 in Study 1.

#### Demographics

Age and gender were included in this study because these employee characteristics may relate to motivational outlooks at work (cf. Gagné et al., 2015). Both demographics questions used in Study 1 were part of a pre-existing pool of items regularly launched by the participating organization, so researchers were unable to alter their format. Age in Study 1 was measured using a single survey item that asked for respondents to indicate the year they were born by selecting among ranges of years. Some categories of age had too few respondents for analyses to be stable, so empirical criteria were used to dichotomize the age variable (born in 1960 or earlier vs. born in 1961 or later) in preparation for analysis. Gender was measured by asking respondents if they were male or female.

### Participants for Study 2

An invitation to participate in the study was sent electronically to a listserv of ~40,000 employees across the United States. The sample of participants included 1,103 employees of various organizations, or a 3% response rate. Females made up 58% of the respondents. Data were not available on participants' ethnic or educational backgrounds, but 67% percent were from organizations operating within the United States. Thirty-three percent of respondents were non-managers, with an average age of 49. Forty-one percent had been with their current organization for more than 10 years, 55% had been in their current position for 4 years or less, and 70% had been reporting to their current supervisor for 4 years or less. Thirty-three percent worked for organizations with 500 or fewer employees, and 20% were from organizations with more than 20,000 employees.

### Procedures for Study 2

Access to the listserv was granted by an international training and management company that works with organizations from various industries. Respondents were granted access to the company's white papers as an incentive for their participation.

#### Measures

For the second study, we used the same measures for power, motivation, and work intentions as we did in the first study (i.e., the IPI, MWMS, and WII). In Study 2, alpha reliabilities for the power subscales ranged from 0.84 to 0.93, and alpha reliabilities for the motivation subscales ranged from 0.70 to 0.89—notable improvements from reliability issues observed from these subscales in Study 1. Specifically, in Study 2, the reliability of amotivation and introjected regulation was acceptable at 0.85 and 0.70, respectively. Also, in Study 2 a lower percentage of respondents reported being “not at all” amotivated at work (i.e., 67–72% for the three amotivation items compared to 81–89% in Study 1), so for Study 2 the amotivation subscale was not dichotomized and was instead calculated from the amotivation items in their original, continuous format. In Study 2, alpha reliabilities for the work intentions subscales ranged from 0.73 to 0.95.

#### Demographics

To measure respondent demographics, age and gender were again included in Study 2. In this launch, we had the option to redesign the age variable to better capture variability in respondents; thus, instead of the categorical, generationally-based scale for age used in Study 1, in Study 2 respondents were asked to type their age in years. No additional data manipulation was conducted on our age variable in the second study prior to analysis because it was analyzed as a continuous variable in our regression analyses in Study 2.

Study 2 used a larger sample spanning organizations across North America, and provided improved reliability coefficients for amotivation and introjected regulation compared to Study 1.

### Evaluating Common Method Bias

We aimed to examine intrapersonal psychological phenomena for this work, so we collected self-report data. Self-report data is most appropriate for learning about respondents' inner experiences and perceptions, which were of focal interest here. Specifically, Chan (2009) highlighted the valuable insight self-report data may provide for researchers aiming to investigate potential affective, cognitive, and motivational processes implicated with individuals' response patterns. Some researchers have expressed concern about the possibility of relationship inflation among substantive constructs in self-report studies incorporating one source of data only (Spector, 2006), and the potential for such constructs to vary due to significant common method bias effects (Podsakoff and Organ, 1986; Podsakoff et al., 2003). We implemented the following approaches to minimize the likelihood of having problems with common method bias. First, we used measures featuring different kinds of response scales, which helps prevent participants from being overly consistent or automatic in their answers. Specifically, participants were asked to rate on a 7-point scale their perceived reasons underlying their manager's behavior, and then they were asked to reflect on their job experiences using a 6-point and 7-point scale, each involving different response anchors. Second, in separate sections of the survey, we varied the instructions and referent for phenomena being rated (i.e., participants were asked to shift from rating their manager's behavior prior to rating their own motivations and intentions on the job).

To additionally probe for the negative effects of common method bias, we applied Harman's single factor procedure (Podsakoff and Organ, 1986; Podsakoff et al., 2003). While this technique for evaluating issues with common method variance may be limited, if only one factor accounts for the majority of variance in the data, that could indicate problems attributable to common method bias (Podsakoff et al., 2003). In both studies, we ran Harman's procedure using exploratory factor analysis. Four and three factors were evident in Study 1 and Study 2, respectively, whereby the first factor accounted for 21.7% (Study 1) and 24.1% (Study 2) of the variance. We also, in both studies, used the marker variable approach suggested by Williams et al. (2010), which involves testing five nested models. In both studies, we found: (1) our data fit the Method-U model better than the Method-C model (i.e., our marker was differentially associated with our variables of interest), and (2) our data did not fit significantly better for the Method-R model than the Method-U model, which indicated that common method bias was not a problem. Harman's procedure and the Williams et al. (2010) both provided evidence that issues with common method bias were not evident in either study.

## Path Analysis Approach

We used Mplus 7.2 and maximum likelihood estimation (MLR) to run competing multivariate models to test our conceptual model involving leader power use, followers' motivational outlooks, and followers' work intentions (i.e., Hypotheses 1a−1d, 2a−2b). Initially we attempted to conduct the full analysis as structural equation models by using latent variables for all substantive constructs, but for the smaller dataset in Study 1, we had too many parameters of interest to estimate, relative to our available statistical power. We therefore modeled both samples using path analysis, which only included two latent variables, one for soft power and one for hard power, and all other key variables were observed scale scores.

At the measurement model level, hard power as a latent variable was calculated from three mean scale scores: reward power, coercive power, and legitimate power. Our latent variable for soft power was calculated from the following three mean scale scores: expert, referent, informational power. Measurement model fit for the power variables was not adequate initially, but model modification indices showed that coercive power also should cross-load onto soft power. Upon making this modification, model fit improved (Δχ^2^[1] = 40.28, *p* < 0.001) and was well-fitting to the data (χ^2^[7] = 57.527, CFI = 0.902, SRMR = 0.060, RMSEA = 0.182). The cross-loading of coercive power onto soft power was −0.544 in Study 1.

Because we anticipated certain kinds of motivational outlooks would be highly related, we allowed them to correlate: i.e., external and introjected regulation were correlated, and so were identified and intrinsic motivation. In accordance with theory and previous research conducted on work intentions, we also specified correlations among the five work intentions. All path models controlled for respondent age and gender.

Overall model fit was evaluated using the following indices: the comparative fit index (CFI), the root mean square error of approximation (RMSEA), and the standardized root mean square residual (SRMR). Specifically, we retained models demonstrating CFI values >0.90, RMSEA values < 0.06, and SRMR values < 0.08 (Hu and Bentler, 1999; Hooper et al., 2008). Chi-square difference testing compared the fit of nested models for full and partial mediation. In each study, path analysis began by running a full mediation model in accordance with our overall conceptual model (Model 1), and that was followed by running variations of partial mediation models, whereby: starting with the full mediation model, five direct paths were added from hard power to each of the work intentions (Model 2); then starting again with the full mediation model, five direct paths were added from soft power to every work intention (Model 3); then all significant direct paths from Model 2 and Model 3 were noted so they could be added collectively to the full mediation model (Model 4); and then Model 4 was examined for non-significant direct paths so they could be removed for subsequent partial mediation model testing (Models 5–6).

## Study 1 Path Analysis Results

**Figure 2:**
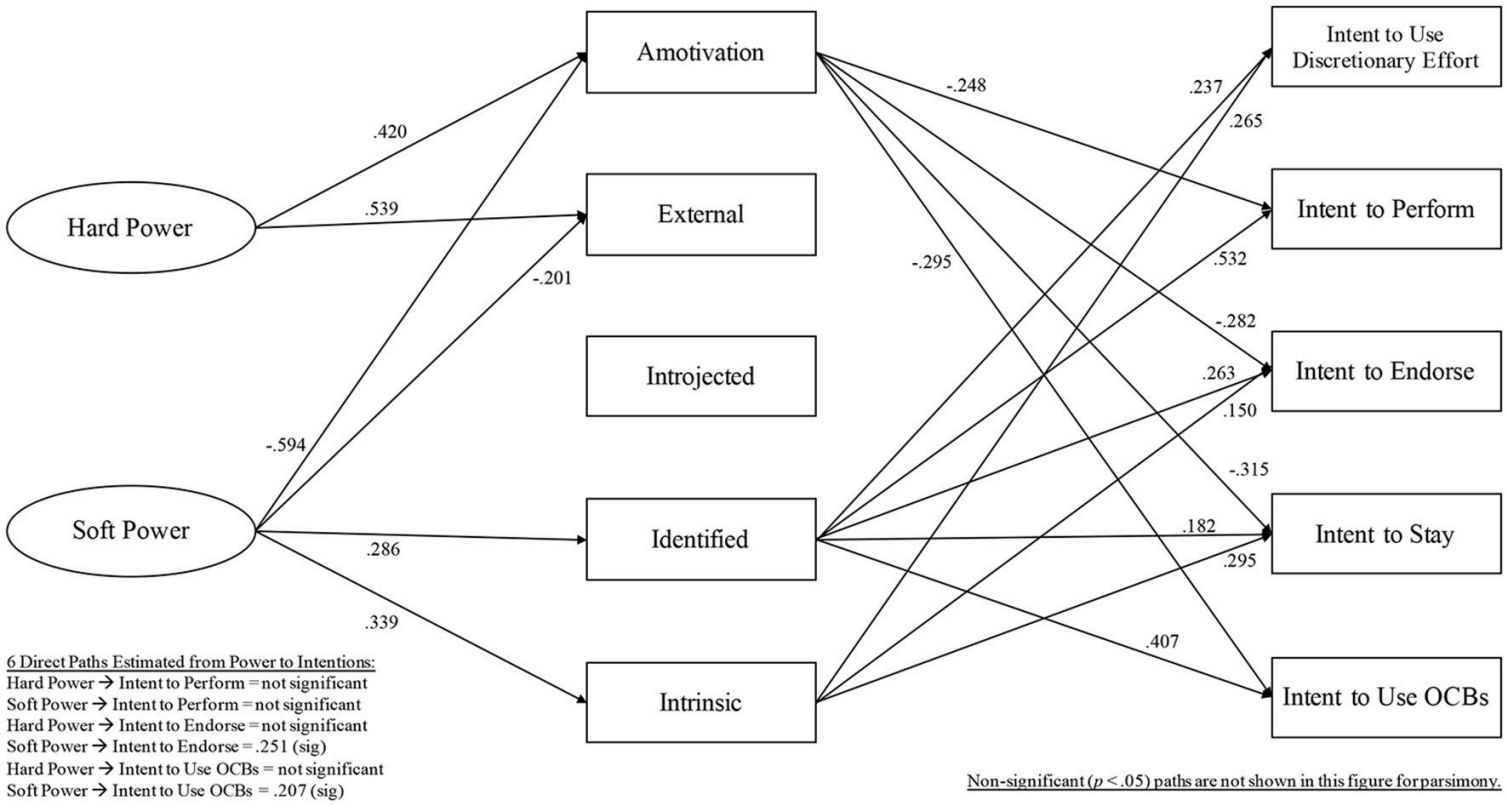
Model 4, Final Model, Study 1 (*n* = 215).

Table 1 provides Study 1 variables' means, standard deviations, correlations, and alpha reliabilities. In Study 1, Models 1–3 were run as described above. Then, in Model 4, a partial mediation model was run by adding 6 direct paths: hard power to intent to perform, soft power to intent to perform, hard power to intent to endorse, soft power to intent to endorse, hard power to intent to use OCBs, and soft power to intent to use OCBs. Compared to Model 1, Models 2–4 fit the data better (Model 2: Δχ^2^[5] = 11.90, *p* < 0.05; Model 3: Δχ^2^[5] = 21.58, *p* < 0.001; Model 4: Δχ^2^[6] = 21.72, *p* < 0.01). Model 5 removed the non-significant direct paths found in Model 4, and only included 2 direct paths: soft power to intent to endorse, and soft power to intent to use OCBs. Model 5 was compared to Model 4 and did not fit the data better (Δχ^2^[4] = 8.72, *p* > 0.05). Results from chi-square significance testing to compare nested models is included in Table 2. Model 4, shown in Figure 2, fit the data best.

**Table 1:** Study 1—scale score means, standard deviations, reliabilities, and correlations.

	**Mean**	**SD**	**1**	**2**	**3**	**4**	**5**	**6**	**7**	**8**	**9**	**10**	**11**	**12**	**13**
(1) Amotivation	1.14	0.24	(0.51)												
(2) External regulation	3.29	1.19	0.112	(0.80)											
(3) Introjected regulation	4.53	1.21	−0.103	0.486^**^	(0.65)										
(4) Identified regulation	6.25	0.77	−0.250^**^	−0.022	0.309^**^	(0.83)									
(5) Intrinsic motivation	5.57	1.12	−0.218^**^	−0.043	0.151^*^	0.569^**^	(0.90)								
(6) Reward power	3.79	1.56	−0.006	0.428^**^	0.228^**^	−0.028	−0.005	(0.87)							
(7) Coercive power	2.99	1.58	0.248^**^	0.379^**^	0.098	−0.029	−0.089	0.624^**^	(0.88)						
(8) Legitimate power	3.65	1.13	0.064	0.383^**^	0.200^**^	−0.032	−0.097	0.694^**^	0.674^**^	(0.87)					
(9) Expert power	4.32	1.77	−0.170^*^	0.087	0.198^**^	0.123	0.142^*^	0.288^**^	0.065	0.251^**^	(0.95)				
(10) Referent power	4.82	1.59	−0.192^**^	0.131	0.152^*^	0.084	0.109	0.551^**^	0.145^*^	0.432^**^	0.484^**^	(0.89)			
(11) Informational power	5.69	1.32	−0.298^**^	0.034	0.107	0.201^**^	0.153^*^	0.232^**^	−0.022	0.244^**^	0.596^**^	0.447^**^	(0.96)		
(12) Gender	1.70	0.46	−0.148^*^	0.071	0.069	0.198^**^	−0.011	0.053	0.090	0.019	0.092	0.032	0.216^**^	(–)	
(13) Age	1.40	0.49	−0.089	−0.193^**^	−0.076	0.113	0.241^**^	−0.240^**^	−0.106	−0.085	−0.082	−0.174^*^	0.048	0.119	(–)

*Cronbach's alpha estimates are in parentheses on the diagonal. Pairwise deletion, ns = 216–228. Gender coded as 1 = male, 2 = female. Age coded as 1 = born 1961 or later, 2 = born 1960 or earlier*.

** *Correlation is significant at the 0.01 level*.

* *Correlation is significant at the 0.05 level*.

**Table 2:** Chi-square significance testing for comparison of structural equation model fit.

**Study number**	**Model number**	**Model description**	**CFI**	**SRMR**	**RMSEA**	**χ^**2**^**	**df**	**Δ in χ^**2**^**	**df Difference**	**Sig**	**Fit notes**
1	Model 1	Hypothesized full mediation model	0.919	0.07	0.083	190.161	77				No comparison.
1	Model 2	Partial mediation model with 5 paths added, hard power to intentions	0.924	0.062	0.083	178.263	72	11.90	5	< 0.05	Comparing Model 2 with Model 1.
1	Model 3	Partial mediation model with 5 paths added, soft power to intentions	0.931	0.059	0.079	168.578	72	21.58	5	< 0.001	Comparing Model 3 with Model 1.
1	Model 4	Partial mediation model with 6 paths added	0.93	0.06	0.08	168.446	71	21.72	6	< 0.01	Comparing Model 4 to Model 1. Best Fitting Model.
1	Model 5	Partial mediation model with 2 paths added	0.927	0.065	0.08	177.161	75	8.72	4	>0.05	Comparing Model 5 to Model 4.

All standardized path coefficients for Model 4 are shown in Figure 2, and respective endogenous variables' *r*^2^ values were as follows: 0.25 for amotivation, 0.23 for external regulation, 0.072 for introjected regulation, 0.11 for identified regulation, 0.18 for intrinsic motivation, 0.23 for intent to use discretionary effort, 0.42 for intent to perform, 0.39 for intent to endorse, 0.35 for intent to stay, 0.37 for intent to use OCBs. Of the above listed *r*^2^ values, only introjected regulation was not significant (*p* > 0.05). Table 3 reports significant indirect effects.

**Table 3:** Summary of significant, specific indirect effects for Model 4 in Study 1.

**Path**	**Indirect effect**	**SE**
**Hard power → …**		
Amotivation → IP	−0.104	0.045
Amotivation → IE	−0.118	0.044
Amotivation → IS	−0.132	0.044
Amotivation → IOCB	−0.124	0.042
**Soft power → …**		
Intrinsic → IDE	0.090	0.039
Amotivation → IP	0.147	0.013
Identified → IP	0.152	0.061
Amotivation → IE	0.167	0.059
Identified → IE	0.075	0.035
Amotivation → IS	0.187	0.065
Intrinsic → IS	0.100	0.039
Amotivation → IOCB	0.175	0.056
Identified → IOCB	0.116	0.052

*Only significant indirect effects are shown, p < 0.05*.

## Study 2 Path Analysis Results

Variables' means, standard deviations, correlations, and alpha reliabilities for Study 2 are shown in Table 4. Similar to Study 1, in Study 2 power variables' measurement model fit to the data was improved by allowing coercive power to load onto soft power (Δχ^2^[1] = 263.72, *p* < 0.001), resulting in a well-fitting measurement model for power (χ^2^[7] = 42.442, CFI = 0.981, SRMR = 0.025, RMSEA = 0.068). In Study 2, coercive power loaded onto soft power at −0.662. We ran structural Models 1–3 in the same way as previously presented in Study 1.

**Table 4:** Study 2—scale score means, standard deviations, reliabilities, and correlations.

	**Mean**	**SD**	**1**	**2**	**3**	**4**	**5**	**6**	**7**	**8**	**9**	**10**	**11**	**12**	**13**
(1) Amotivation	1.60	1.03	(0.85)												
(2) External regulation	3.32	1.14	0.199^**^	(0.80)											
(3) Introjected regulation	4.51	1.20	−0.027	0.427^**^	(0.70)										
(4) Identified regulation	5.89	0.97	−0.399^**^	−0.019	0.331^**^	(0.83)									
(5) Intrinsic motivation	5.30	1.20	−0.374^**^	−0.033	0.222^**^	0.642^**^	(0.89)								
(6) Reward power	3.81	1.42	0.047	0.439^**^	0.269^**^	0.095^**^	0.112^**^	(0.84)							
(7) Coercive power	3.41	1.46	0.226^**^	0.465^**^	0.211^**^	−0.064^*^	−0.126^**^	0.550^**^	(0.85)						
(8) Legitimate power	3.67	1.02	0.121^**^	0.337^**^	0.249^**^	0.085^**^	0.084^**^	0.619^**^	0.455^**^	(0.85)					
(9) Expert power	4.16	1.67	−0.103^**^	0.037	0.131^**^	0.190^**^	0.233^**^	0.264^**^	−0.034	0.325^**^	(0.93)				
(10) Referent power	4.49	1.55	−0.187^**^	0.096^**^	0.187^**^	0.282^**^	0.304^**^	0.428^**^	−0.008	0.471^**^	0.587^**^	(0.85)			
(11) Informational power	5.73	1.31	−0.219^**^	0.042	0.182^**^	0.294^**^	0.281^**^	0.302^**^	−0.026	0.290^**^	0.466^**^	0.496^**^	(0.93)		
(12) Gender	1.70	0.46	−0.047	0.053	0.080^**^	0.042	−0.051	0.023	0.072^*^	−0.028	0.019	0.008	0.071^*^	(–)	
(13) Age	1.60	0.49	−0.088^**^	−0.193^**^	−0.151^**^	0.166^**^	0.133^**^	−0.086^**^	−0.071^*^	−0.039	−0.054	−0.018	0.005	−0.128^**^	(–)

*Cronbach's alpha estimates are in parentheses on the diagonal. Pairwise deletion, ns = 1,095–1,103. Gender coded as 1 = male, 2 = female. Age was a continuous variable with lower values representing younger ages*.

** *Correlation is significant at the 0.01 level*.

* *Correlation is significant at the 0.05 level*.

**Figure 3:**
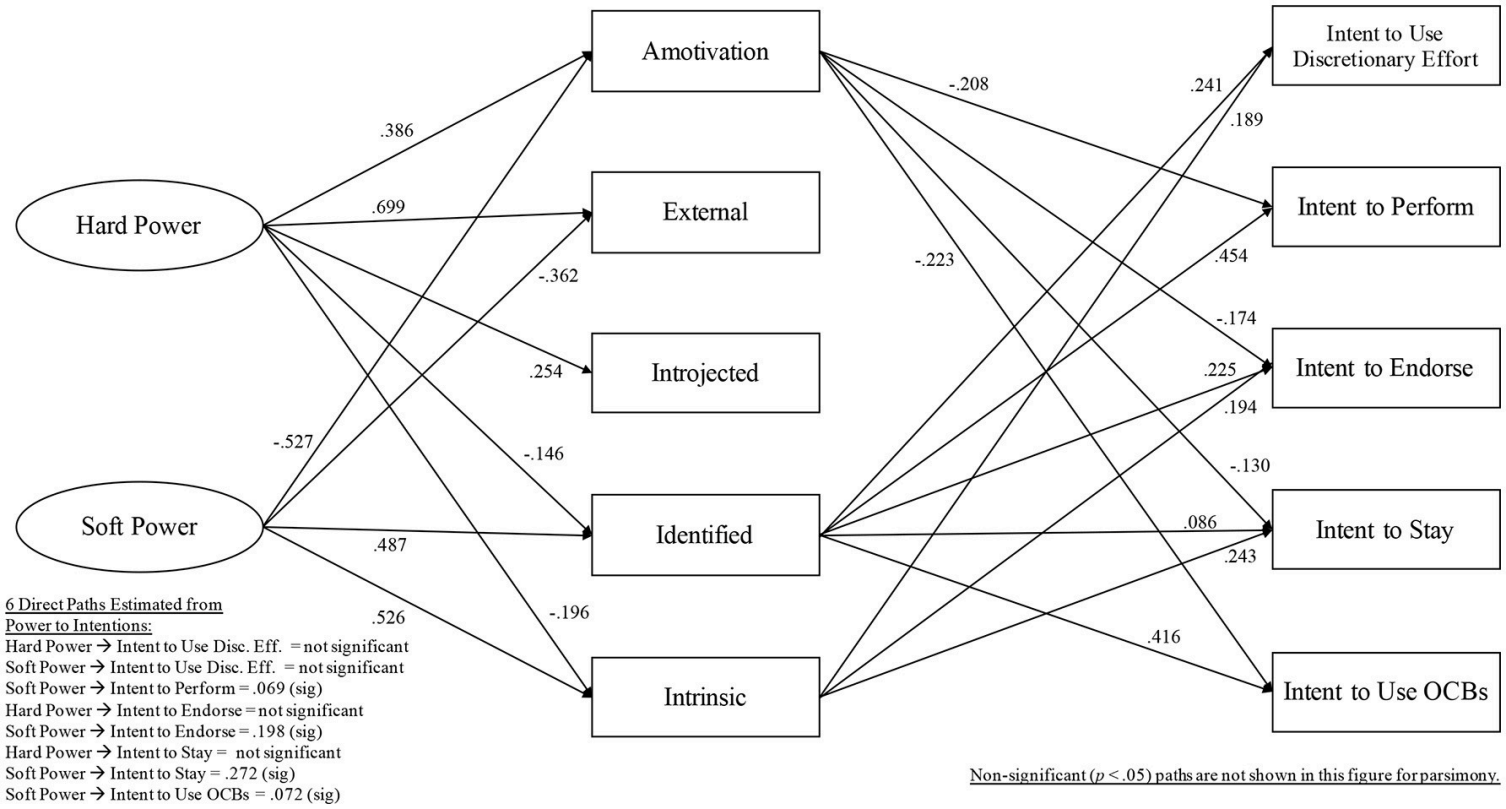
Model 4, Final Model, Study 2 (*n* = 1,039).

Model 4 was a partial mediation model with the following 8 direct paths added: hard power to intent to use discretionary effort, soft power to intent to use discretionary effort, soft power to intent to perform, hard power to intent to endorse, soft power to intent to endorse, hard power to intent to stay, soft power to intent to stay, soft power to intent to use OCBs. Relative to Model 1, Models 2–4 fit the data better (Model 2: Δχ^2^[5] = 12.05, *p* < 0.05; Model 3: Δχ^2^[5] = 37.91, *p* < 0.001; Model 4: Δχ^2^[8] = 41.89, *p* < 0.001). Removing the non-significant direct paths from Model 4, Model 5 included 4 direct paths: soft power to intent to perform, soft power to intent to endorse, soft power to intent to stay, soft power to intent to use OCBs. Model 5 did not fit the data significantly better than Model 4 (Δχ^2^[4] = 8.78, *p* > 0.05). Applying the same logic, Model 6 included only 2 direct paths: soft power to intent to endorse, soft power to intent to stay. Model 6 did not show better fit to the data than Model 4 (Δχ^2^[6] = 11.19, *p* > 0.05). See Table 5 for model comparison results from chi-square significance testing, and Figure 3 for Model 4, which was best-fitting to the data.

**Table 5:** Chi-square significance testing for comparison of structural equation model fit.

**Study number**	**Model number**	**Model description**	**CFI**	**SRMR**	**RMSEA**	**χ^**2**^**	**df**	**Δ in χ^**2**^**	**df Difference**	**Sig**	**Fit notes**
2	Model 1	Hypothesized full mediation model	0.933	0.057	0.071	475.786	77				No comparison.
2	Model 2	Partial mediation model with 5 paths added, hard power to intentions	0.934	0.054	0.072	463.738	72	12.05	5	< 0.05	Comparing Model 2 with Model 1.
2	Model 3	Partial mediation model with 5 paths added, soft power to intentions	0.939	0.052	0.07	437.879	72	37.91	5	< 0.001	Comparing Model 3 with Model 1.
2	Model 4	Partial mediation model with 8 paths added	0.939	0.052	0.071	433.894	69	41.89	8	< 0.001	Comparing Model 4 to Model 1. Best Fitting Model.
2	Model 5	Partial mediation model with 4 paths added	0.938	0.053	0.07	442.679	73	8.78	4	>0.05	Comparing Model 5 to Model 4.
2	Model 6	Partial mediation model with 2 paths added	0.938	0.054	0.069	445.08	75	11.19	6	>0.05	Comparing Model 6 with Model 4.

For Model 4, standardized path coefficients are presented in Figure 3. For that final model, endogenous variables' *r*^2^ values were: 0.18 for amotivation, 0.33 for external regulation, 0.13 for introjected regulation, 0.20 for identified regulation, 0.21 for intrinsic motivation, 0.20 for intent to use discretionary effort, 0.35 for intent Ω to perform, 0.32 for intent to endorse, 0.30 for intent to stay, 0.27 for intent to use OCBs. Of the above listed *r*^2^ values, all were significant (*p* < 0.001). Significant indirect effects are shown in Table 6.

**Table 6:** Summary of significant, specific indirect effects for Model 4 in Study 2.

**Path**	**Indirect Effect**	**SE**
**Hard power → …**		
Identified → IDE	−0.035	0.014
Intrinsic → IDE	−0.037	0.012
Amotivation → IP	−0.080	0.015
Identified → IP	−0.066	0.024
Amotivation → IE	−0.067	0.014
Identified → IE	−0.033	0.013
Intrinsic → IE	−0.038	0.012
Amotivation → IS	−0.050	0.014
Intrinsic → IS	−0.048	0.014
Amotivation → IOCB	−0.086	0.018
Identified → IOCB	−0.061	0.022
**Soft power →**		
Identified → IDE	0.117	0.026
Intrinsic → IDE	0.099	0.023
Amotivation → IP	0.110	0.021
Identified → IP	0.221	0.032
Amotivation → IE	0.092	0.019
Identified → IE	0.109	0.024
Intrinsic → IE	0.102	0.022
Amotivation → IS	0.068	0.018
Identified → IS	0.042	0.018
Intrinsic → IS	0.128	0.023
Amotivation → IOCB	0.117	0.023
Identified → IOCB	0.203	0.030

*Only significant indirect effects are shown, p < 0.05*.

## Studies 1 And 2: Interpretation Of Final Models

*Hypothesis 1a* was mostly supported by both studies' final models, leaders' use of hard power was strongly and positively correlated with followers' amotivation and external regulation (sub-optimal motivational outlooks). For the path from hard power to amotivation, in Study 1 β = 0.42, *p* < 0.05, and in Study 2, β = 0.39, *p* < 0.05. Similarly, hard power was related to external regulation: Study 1 β = 0.54, *p* < 0.05, and in Study 2, β = 0.70, *p* < 0.05. A significant path between hard power and introjected regulation was only found in Study 2, however, possibly due to having more statistical power in that sample (β = 0.25, *p* < 0.05). Followers who perceived greater hard power use by their leaders were more likely to hold higher levels of sub-motivational outlooks at work.

*Hypothesis lb* was supported by the final model of Study 1, which found no significant relationships between leaders' use of hard power and identified or intrinsic motivation. In Study 2, this hypothesis was supported in that there were negative relationships between leaders' use of hard power and identified (β = −0.15, *p* < 0.05) and intrinsic (β = −0.20, *p* < 0.05) motivation. Thus, followers with leaders exerting higher levels of hard power use were somewhat more likely to report lower levels of optimal motivation.

In both studies, *Hypothesis 1c* was supported. Leaders' use of soft power was significantly and negatively related to followers' amotivation (β = −0.59, *p* < 0.05 in Study 1, and β = −0.53, *p* < 0.05 in Study 2) and to followers' external regulation (β = −0.20, *p* < 0.05 in Study 1, and β = −0.36, *p* < 0.05 in Study 2). In both studies, soft power was not significantly related to followers' introjected regulation. Followers who viewed higher amounts of soft power use by their leaders were more likely to report lower levels of amotivation and lower levels of external regulation.

*Hypothesis 1d* was supported by both studies, as soft power use by leaders positively correlated with followers' optimal motivational outlooks. Leaders' use of soft power was significantly related to followers' identified regulation (β = 0.29, *p* < 0.05 in Study 1, and β = 0.49, *p* < 0.05 in Study 2) and to followers' intrinsic motivation (β = 0.34, *p* < 0.05 in Study 1, and β = 0.53, *p* < 0.05 in Study 2). Followers who perceived higher soft power use from their leaders were more likely to report higher levels of optimal motivation.

Evidence supporting *Hypothesis 2a* was found in both studies, as followers' sub-optimal motivational outlooks (i.e., amotivation, external regulation, introjected regulation) either negatively correlated with, or did not correlate with, their work intentions. Four of five paths from amotivation to work intentions were negative and significant (i.e., for intent to perform β = −0.25, *p* < 0.05 in Study 1, and β = −0.21 *p* < 0.05 in Study 2; for intent to endorse β = −0.28, *p* < 0.05 in Study 1, and β = −0.17 *p* < 0.05 in Study 2; for intent to stay β = −0.32, *p* < 0.05 in Study 1, and β = −0.13, *p* < 0.05 in Study 2; for intent to use OCBs β = −0.30, *p* < 0.05 in Study 1, and β = −0.22, *p* < 0.05 in Study 2). Therefore, followers experiencing greater levels of amotivation were more likely to score lower on four of the five work intentions. Paths between amotivation and intent to use discretionary effort were not significant in either study. External regulation and introjected regulation were not significantly related to any of the work intentions in either study.

*Hypothesis 2b* was supported by both studies in eight of ten possible paths: for the most part, followers' optimal motivational outlooks (i.e., identified, intrinsic motivation) positively and significantly correlated with their work intentions. Identified regulation was positively related to the all five work intentions in Study 1 (βs ranged from 0.18 to 0.53, *p* < 0.05) and in Study 2 (βs ranged from 0.09 to 0.45, *p* < 0.05). Positive and significant paths were also found from intrinsic motivation to intent to use discretionary effort (β = 0.27, *p* < 0.05 in Study 1, and β = 0.19, *p* < 0.05 in Study 2), to intent to endorse (β = 0.15, *p* < 0.05 in Study 1, and β = 0.19 *p* < 0.05 in Study 2) and to intent to stay (β = 0.30, *p* < 0.05 in Study 1, and β = 0.24 *p* < 0.05 in Study 2). Taken together, except for intent to perform and intent to use OCBs, followers with higher levels of optimal motivation were more likely to report favorable levels of work intentions.

In both studies, partial mediation models fit the data better than complete mediation models, thereby somewhat supporting *Hypothesis 3a*. Finally, regarding direct paths from power to work intentions, followers who perceived their leaders used higher levels of soft power were more likely to intend to endorse their organizations (β = 0.25, *p* < 0.05 in Study 1, and β = 0.20, *p* < 0.05 in Study 2) and to intend to use OCBs (β = 0.21, *p* < 0.05 in Study 1, and β = 0.07, *p* < 0.05 in Study 2). In Study 2 only, followers working under leaders who used soft power reported increased intentions to stay with their organization (β = 0.27, *p* < 0.05).

## Regression Approach

As a follow-up analysis to supplement our path modeling, we used hierarchical multiple regression analysis in SPSS version 22, which tested *Hypotheses 4a* and *4b* to explain variance in the five motivational outlooks and the five work intentions. Specifically, we were interested in evaluating the potential effects of followers' experiences of their leaders' use of multiple kinds of hard or soft power in combination with one another.

In preparation for this analysis, in each study's dataset, we transformed power use so it could be meaningfully aggregated to indicate followers' perceptions of the degree of each kind of power was being used by their leader. As the subscale for the power measures ranged from 1 (to no extent) to 6 (to the fullest extent), we examined the labels of the response scales as well as the distributions of responses in each study, to determine that values of 4.5 or higher would allow for the meaningful dichotomization of followers' perceptions of their managers. That is, for every power variable, we re-coded followers with ratings of 4.49 and lower (i.e., the lower half of the “to a great extent” anchor and including all responses through “to no extent”) as 1s, and we re-coded followers with power ratings of 4.5 and higher (i.e., the upper half of the “to a great extent” anchor and including responses indicating “to a very great extent” and “to the fullest extent”) as 2s. Then, for each respondent, we summed the recoded scores on all hard power variables (coercive, reward, legitimate), to create a single variable that indicated the degree of hard power used by their leader, and aggregating across all types of hard power. Thus, scores for this new sum combination variable for hard power ranged from 3 to 6, with 3 indicating that a follower's leader uses low levels of all kinds of hard power, and 6 indicating that a follower's leader uses high levels of all kinds of hard power. The same transformation was done for soft power, prior to regression analysis.

In the regression analyses, respondent age and gender were entered into Step 1, and the two sum combination variables for hard and soft power were then also entered into Step 2. The mean imputation procedure within SPSS was conducted for cases with missing data.

## Studies 1 And 2: Regression Results And Interpretation

Follow-up regression results are shown in Table 7 for Study 1, and in Table 8 for Study 2. *Hypothesis 4a* was supported in both studies, as followers of leaders who used multiple kinds of hard power at high levels demonstrated higher levels of amotivation (β = 0.16, *p* < 0.05 in Study 1, and β = 0.18, *p* < 0.05 in Study 2), external regulation (β = 0.41, *p* < 0.05 in Study 1, and β = 0.44, *p* < 0.05 in Study 2), and introjected regulation (β = 0.15, *p* < 0.05 in Study 1, and β = 0.23, *p* < 0.05 in Study 2). When leaders engaged in using many kinds of hard power, sub-optimal motivation levels in followers were more pronounced. Additionally, *Hypothesis 4a* was also confirmed in both studies as no significant relationships were found between followers' perceptions of their leaders' use of multiple kinds of hard power and follower's optimal motivational outlooks, or followers' work intentions.

**Table 7:** Study 1—combinations of power regression models, controlling for age and gender (*n* = 229).

	**Amotivation**	**External**	**Introjected**	**Identified**	**Intrinsic**	**IDE**	**IP**	**IE**	**IS**	**IOCB**
	**β**	**β**	**β**	**β**	**β**	**β**	**β**	**β**	**β**	**β**
**STEP 1**										
Age (1 = born 1961 or later; 2 = born 1960 or earlier)	−0.073	−0.204*	−0.084	0.090	0.242*	0.060	−0.005	−0.013	0.227*	−0.031
Gender (1 = male, 2 = female)	−0.132*	0.093	0.077	0.183*	−0.039	−0.106	0.092	0.012	−0.098	0.092
**STEP 2**										
Hard power combo sum	0.161*	0.405*	0.154*	−0.064	−0.069	−0.011	0.024	0.003	−0.054	−0.009
Soft power combo sum	−0.307*	−0.041	0.128	0.147*	0.188*	−0.008	0.194*	0.311*	0.295*	0.273*
**Model summary statistics**										
**Dependent variable**	**Step 1**					**Step 2**				
Amotivation	*F*_(2, 226)_ = 2.90, *p* > 0.05, *R*^2^ = 0.025					*F*_(4, 224)_ = 7.57, *p* < 0.001, *R*^2^ = 0.119; Δ*R*^2^ = 0.094				
External	*F*_(2, 226)_ = 5.43, *p* < 0.01, *R*^2^ = 0.046					*F*_(4, 224)_ = 14.20, *p* < 0.001, *R*^2^ = 0.202; Δ*R*^2^ = 0.156				
Introjected	*F*_(2, 226)_ = 1.32, *p* > 0.05, *R*^2^ = 0.012					*F*_(4, 224)_ = 3.63, *p* < 0.01, *R*^2^ = 0.061; Δ*R*^2^ = 0.049				
Identified	*F*_(2, 226)_ = 5.39, *p* < 0.01, *R*^2^ = 0.046					*F*_(4, 224)_ = 3.97, *p* < 0.01, *R*^2^ = 0.066; Δ*R*^2^ = 0.021				
Intrinsic	*F*_(2, 226)_ = 6.96, *p* < 0.01, *R*^2^ = 0.058					*F*_(4, 224)_ = 5.61, *p* < 0.001, *R*^2^ = 0.091; Δ*R*^2^ = 0.033				
IDE	*F*_(2, 226)_ = 1.54, *p* > 0.05, *R*^2^ = 0.013					*F*_(4, 224)_ = 0.78, *p* > 0.05, *R*^2^ = 0.014; Δ*R*^2^ = 0.001				
IP	*F*_(2, 226)_ = 0.96, *p* > 0.05, *R*^2^ = 0.008					*F*_(4, 224)_ = 2.84, *p* < 0.05, *R*^2^ = 0.048; Δ*R*^2^ = 0.040				
IE	*F*_(2, 226)_ = 0.032, *p* > 0.05, *R*^2^ = 0.001					*F*_(4, 224)_ = 5.92, *p* < 0.001, *R*^2^ = 0.096; Δ*R*^2^ = 0.095				
IS	*F*_(2, 226)_ = 6.68, *p* < 0.01, *R*^2^ = 0.056					*F*_(4, 224)_ = 8.83, *p* < 0.001, *R*^2^ = 0.136; Δ*R*^2^ = 0.080				
IOCB	*F*_(2, 226)_ = 1.01, *p* > 0.05, *R*^2^ = 0.009					*F*_(4, 224)_ = 4.95, *p* < 0.01, *R*^2^ = 0.081; Δ*R*^2^ = 0.072				

*β value shows asterisk if p < 0.05. Higher values for age represent older respondents*.

**Table 8:** Study 2—combinations of power regression models, controlling for age and gender (*n* = 1,103).

	**Amotivation**	**External**	**Introjected**	**Identified**	**Intrinsic**	**IDE**	**IP**	**IE**	**IS**	**IOCB**
	**β**	**β**	**β**	**β**	**β**	**β**	**β**	**β**	**β**	**β**
**STEP 1**										
Age	−0.092*	−0.183*	−0.138*	0.165	0.123*	0.039	0.077*	0.105*	0.164*	0.120*
Gender (1 = male, 2 = female)	−0.059	0.030	0.062	0.061*	−0.035	−0.050	0.110*	−0.022	−0.052	0.104*
**STEP 2**										
Hard power combo sum	0.177*	0.440*	0.228*	0.016	−0.032	0.058	−0.006	−0.004	−0.051	−0.022
Soft power combo sum	−0.218*	−0.026	0.123*	0.264*	0.310*	0.184*	0.251*	0.292*	0.269*	0.217*
**Model summary statistics**										
**Dependent variable**	**Step 1**					**Step 2**				
Amotivation	*F*_(2, 1100)_ = 5.81, *p* < 0.01, *R*^2^ = 0.010					*F*_(4, 1098)_ = 21.46, *p* < 0.001, *R*^2^ = 0.073; Δ*R*^2^ = 0.062				
External	*F*_(2, 1100)_ = 20.35, *p* < 0.001, *R*^2^ = 0.036					*F*_(4, 1098)_ = 79.65, *p* < 0.001, *R*^2^ = 0.223; Δ*R*^2^ = 0.188				
Introjected	*F*_(2, 1100)_ = 14.07, *p* < 0.001, *R*^2^ = 0.025					*F*_(4, 1098)_ = 31.74, *p* < 0.001, *R*^2^ = 0.104; Δ*R*^2^ = 0.079				
Identified	*F*_(2, 1100)_ = 16.06, *p* < 0.001, *R*^2^ = 0.028					*F*_(4, 1098)_ = 30.52, *p* < 0.001, *R*^2^ = 0.100; Δ*R*^2^ = 0.072				
Intrinsic	*F*_(2, 1100)_ = 9.73, *p* < 0.001, *R*^2^ = 0.017					*F*_(4, 1098)_ = 34.04, *p* < 0.001, *R*^2^ = 0.110; Δ*R*^2^ = 0.093				
IDE	*F*_(2, 1100)_ = 2.54, *p* > 0.05, *R*^2^ = 0.005					*F*_(4, 1098)_ = 13.30, *p* < 0.001, *R*^2^ = 0.046; Δ*R*^2^ = 0.042				
IP	*F*_(2, 1100)_ = 8.92, *p* < 0.001, *R*^2^ = 0.016					*F*_(4, 1098)_ = 23.27, *p* < 0.001, *R*^2^ = 0.078; Δ*R*^2^ = 0.062				
IE	*F*_(2, 1100)_ = 6.67, *p* < 0.01, *R*^2^ = 0.012					*F*_(4, 1098)_ = 29.31, *p* < 0.001, *R*^2^ = 0.093; Δ*R*^2^ = 0.084				
IS	*F*_(2, 1100)_ = 18.00, *p* < 0.001, *R*^2^ = 0.032					*F*_(4, 1098)_ = 30.79, *p* < 0.001, *R*^2^ = 0.101; Δ*R*^2^ = 0.069				
IOCB	*F*_(2, 1100)_ = 12.45, *p* < 0.001, *R*^2^ = 0.022					*F*_(4, 1098)_ = 19.87, *p* < 0.001, *R*^2^ = 0.067; Δ*R*^2^ = 0.045				

*β value shows asterisk if p < 0.05. Higher values for age represent older respondents*.

In accordance with *Hypothesis 4b*, followers of leaders who used many kinds of soft power at high levels reported higher levels of identified (β = 0.15, *p* < 0.05 in Study 1, and β = 0.26, *p* < 0.05 in Study 2), and intrinsic motivation (β = 0.19, *p* < 0.05 in Study 1, and β = 0.31, *p* < 0.05 in Study 2). Followers with leaders engaging in multiple types of soft power also demonstrated significantly lower amounts of amotivation (β = −0.31, *p* < 0.05 in Study 1, and β = −0.22, *p* < 0.05 in Study 2), no significant change in external regulation (in both studies), and in Study 2 only, somewhat significantly higher levels of introjected regulation (β = 0.12, *p* < 0.05; note, however, the Study 1 β looked similar despite not being significant, which may be due to statistical power). Except for intent to use discretionary effort in Study 1, followers who perceived their leaders used many kinds of soft power were more likely to report favorable levels of work intentions (βs ranged from 0.19 to 0.31, *p* < 0.05 in Study 1, and βs ranged from 0.18 to 0.29, *p* < 0.05 in Study 2). See Table 9 for a summary of all hypotheses and results for both studies.

**Table 9:** Summary of hypotheses and results for studies 1 and 2.

**Hypothesis number**	**Description**	**Study 1**	**Study 2**	**Notes**
1a	Leaders' use of various kinds of hard power will positively correlate with followers' sub-optimal motivation (i.e., amotivation, external regulation, introjected regulation).	Mostly supported	Mostly supported	Significant path between hard power and introjected regulation was only found in Study 2
1b	Leaders' use of various kinds of hard power will negatively correlate with, or not correlate with, followers' optimal motivation (i.e., identified regulation, intrinsic motivation).	Fully supported	Fully supported	Study 1 showed non-significant relationships between hard power and optimal motivation, whereas Study 2 showed negative relationships
1c	Leaders' use of various kinds of soft power will negatively correlate with, or not correlate with, followers' sub-optimal motivation (i.e., amotivation, external regulation, introjected regulation).	Fully supported	Fully supported	Non-significant relationship between soft power and introjected regulation in both studies
1d	Leaders' use of various kinds of soft power will positively correlate with followers' optimal motivation (i.e., identified regulation, intrinsic motivation).	Fully supported	Fully supported	–
2a	Followers' sub-optimal motivation (i.e., amotivation, external regulation, introjected regulation) will negatively correlate with, or not correlate with, their work intentions.	Fully supported	Fully supported	In both studies, paths between amotivation and intent to use discretionary effort were non-significant, as were paths between external/introjected regulation and all work intentions
2b	Followers' optimal motivation (i.e., identified regulation, intrinsic motivation) will positively correlate with their work intentions.	Mostly supported	Mostly supported	In both studies, 8 of 10 paths were positive and significant. The two non-significant paths were between intrinsic motivation and intent to use OCBs, and intrinsic motivation and intent to perform
3a	Followers' motivational outlooks will partially mediate perceptions of leaders' use of various kinds of power and followers' work intentions.	Somewhat supported	Somewhat supported	See Tables 3, 6 for significant indirect effects. Mediation analysis naturally relied on the results of the above listed hypotheses
4a	Followers of leaders who use multiple kinds of hard power at high levels (as compared to leaders who use lower levels of all kinds of hard power) will report higher levels of amotivation, external regulation, and introjected regulation, lower levels of (or no difference in levels of) identified regulation and intrinsic motivation, and lower levels of (or no difference in levels of) work intentions.	Fully supported	Fully supported	–
4b	Followers of leaders who use multiple kinds of soft power at high levels (as compared to leaders who use lower levels of all kinds of soft power) will report higher levels of identified regulation and intrinsic motivation, lower levels of (or no difference in levels of) amotivation, external regulation, and introjected regulation, and higher levels of work intentions.	Mostly supported	Mostly supported	Study 2 showed a positive correlation between the use of multiple kinds of soft power and introjected regulation. Use of multiple kinds of soft power were positively related to work intentions in both studies, with the exception of intent to use discretionary effort in Study 1.

## Discussion

This work calls attention to the types of power that, when used by leaders, are more likely to relate to optimal or sub-optimal motivational outlooks in followers, and varying levels of followers' work intentions. Our findings highlight the benefits of when leaders use soft power with their followers, and the psychological costs to followers when leaders operate from hard power. This study demonstrates SDT is a relevant lens for understanding the psychology of followers working under power, and suggests the value of SDT in organizations.

Importantly, followers who experience their leaders using hard power will be much more likely to have sub-optimal motivational outlooks, particularly amotivation and external regulation. Furthermore, a highly notable finding was that leaders' use of multiple kinds of hard power together will correlate with all types of sub-optimal motivational outlooks in followers, and the strength of this relationship for external regulation is important for practice. This finding aligns with SDT literature and previous research that has provided evidence for negative outcomes associated with the use of hard power in organizations (Podsakoff and Schriesheim, 1985; Elias, 2008; Randolph and Kemery, 2011).

In some cases, followers who perceive their leaders use hard power may also suffer from a slight decline in optimal motivation. Results differed between our studies in the relationship between leaders' hard power use and followers' optimal motivation (i.e., Study 1 found no significant relationships, whereas Study 2 uncovered small negative, significant relationships). We wonder if the findings of Study 2 have more generalizability than Study 1, given that Study 2 was a large sample comprising followers from various organizations, whereas Study 1 was a small sample of followers from a single organization. The observed differences could be due to statistical power, or it is possible that employees from the same organization may collectively experience other factors that could mitigate the psychological sting of hard power. In general, we wonder if the latter may be the case when followers under their leaders' hard power have learned that their leaders still value them overall, as indicated by other cultural norms (e.g., one-on-one lunch outings, expressing the deeper meaning of the work, caring conversations, being granted extra flexibility in their work schedules). Or, perhaps in organizational cultures that normalize autocratic rule from leaders (e.g., military settings), employees may expect to often experience hard power and therefore be less personally affected by it.

Additionally, followers who perceive soft power use from their leaders will be notably more likely to experience optimal motivational outlooks. Followers working with leaders who use higher amounts of soft power may benefit from feeling lower levels of sub-optimal motivation, specifically for amotivation and external regulation. Additionally, when leaders use many kinds of soft power at once, followers' motivational outlooks may benefit by being more optimal, and less characterized by amotivation. The potential compounding psychological effect felt by followers who had leaders using multiple kinds of power was evident in this study, and is in alignment with SDT. Mainly, leaders who exercise many kinds of hard power at once (or many kinds of soft power at once), may be more strongly depleting or enriching followers' basic psychological needs of autonomy, relatedness, and competence than leaders who only use one kind of power.

The quality of followers' motivational outlooks also appears to be connected to followers' intentions to engage in positive work outcomes for their organizations. Amotivation in followers tends to decrease followers' intentions to perform, endorse their organizations, stay with their organizations, and use OCBs, although followers' intentions for discretionary effort may remain unaffected by experiences of amotivation. The other two kinds of followers' sub-optimal motivational outlooks, external regulation and introjected regulation, are not related to followers' work intentions. Conversely, optimal motivational outlooks in followers are very often related to followers' favorable work intentions. These findings strongly support assumptions of SDT, as it is expected that followers who are more optimally motivated will have their basic psychological needs met, and therefore they will have greater capacity to function from a place of strength and resilience at work.

Furthermore, leaders' use of multiple kinds of soft power will relate to the increased likelihood that their followers will intend to work positively for their organization. This relationship was observed in all work intentions variables across both studies, except for in Study 1 where combinations of soft power use did not correlate with followers' intent to use discretionary effort. This may suggest that employees in Study 1, our organizational sample, collectively have other reasons besides soft power to exert strong effort in their work (e.g., recognition, appreciation, teamwork mentality). Also, the final model in Study 2 had a positive and significant direct path between leaders' use of soft power and followers' intentions to stay with their organization, whereas Study 1 did not. Again, the differences in findings could be attributable to statistical power, or something else may be explaining Study 1 followers' variability in their intentions to stay. Qualitatively, we know that employees in the organization used in Study 1 often have high tenure, so it could be that followers' intention to stay with that organization is less related to leader soft power, and perhaps more related to other benefits the company offers.

Overall, the magnitude of the relationships between followers' optimal motivational outlooks and work intentions is relevant for practice; these findings suggest that leading in a manner that encourages others to form and maintain identified and intrinsic motivational outlooks is not only a practice that sounds ideal in principle, but indeed it is a practice that could add great value to workplace outcomes.

Taken together, for leaders who wish to promote sustainable and healthy kinds of motivation in their followers, the above suggests that using soft power is a superior approach to using hard power. Considering the basic tenants SDT, our findings may suggest that leaders' use of hard power will likely disrupt followers' psychological flourishing in the areas of autonomy, relatedness, and competence—and thereby will impair followers' motivation, and their intentions to perform favorably on the job. Thus, we raise the question: are organizational leaders who rule from hard power ultimately undermining themselves?

### Limitations and Recommendations for Future Research

Our findings regarding the introjected motivational outlook were generally inconclusive, so we recommend additional research in that area. It should be noted that Gagné (2016), in a paper presented at the SDT Conference in June of 2016, said that different sub-forms of introjection may be at work in the larger concept of introjection. Gagné offered that the definition of introjection may be incomplete, such that it may include shame and guilt as subconstructs. The nascent state of introjection as an academic concept may be affecting its measurement in our studies and, therefore, may be shaping our results accordingly. It may be worthwhile to investigate whether introjected motivational outlooks may be connected to other context-specific factors besides managerial use of power, or whether perhaps introjected motivational outlooks may instead be more strongly connected to individual personality differences or social axioms held by employees.

Effect decomposition analyses for the path models specifying hard and soft power uncovered total indirect effects between hard power and work intentions, through all motivational outlook variables. Specifically, regarding indirect effects flowing through motivational outlooks, in both studies, employees with managers using higher amounts of hard power were somewhat less likely to report favorable levels of work intentions. The opposite, and slightly stronger, indirect effect was found for employees with managers exercising higher levels of soft power; these employees reported greater intentions to work favorably. Therefore, managerial use of power was related to more than employees' motivational outlooks; productive levels of work intentions were also related to power use. These findings are in keeping with Zigarmi et al. (2016), whose research revealed correlations between amotivation and work intentions (*r*s ranged from −0.14 to −0.31), controlled regulation and work intentions (*r*s ranged from not significant to 0.13), and autonomous regulation and work intentions (*r*s ranged from 0.42 to 0.56). Future research could investigate causal relationships between perceived leader power, employee motivation, and intentions, or other aspects of organizational life that may be influenced by managerial use of certain kinds of power.

Low reliabilities of the amotivation and introjected regulation subscales in Study 1 indicate that conclusions drawn from Study 1 regarding those variables should be made with caution. Also, although demographic effects on motivation differed somewhat between studies, we are optimistic that comparing findings across studies and controlling for demographic differences strengthened our conclusions.

Both studies were convenience samples, so findings may not generalize to broader populations. Additionally, because data were cross-sectional, this study does not provide evidence for the directionality of relationships observed. Thus, future research could examine potential cause-and-effect relationships between leader use of power and forms of employee motivation using longitudinal data. Another potential limitation to this study is the use of single-source, self-report measures. Future research could include objective, observable outcomes to investigate how employee motivation and the use of various forms of leader power might impact organizational performance metrics.

### Conclusion and Practical Implications

Overall, we found that leaders' use of hard power relates to higher levels of sub-optimal motivational outlooks in followers, while leaders' use of soft power is connected to higher levels of optimal motivational outlooks in followers. Followers' motivational outlooks were also related to their intentions to perform favorably for their organizations.

As this work provides insight into the relationship between leaders' use of power and less optimal kinds of follower motivation, we encourage managers in the field to consider how their use of coercive, legitimate, and reward power may be adversely connected to the daily quality of follower motivation. Said differently, leaders who often resort to hard types of power should proceed with caution, because they may unknowingly be undermining their own efforts to inspire an engaging and productive workplace that encourages autonomous regulation. Finally, we encourage both power and non-power holders to carefully consider how any use of power relates to the daily quality of follower motivation.

## Ethics Statement

All procedures performed in studies involving human participants were in accordance with the ethical standards of the institutional and/or national research committee and with the 1964 Helsinki declaration and its later amendments or comparable ethical standards. Namely, the Research Ethics Committee at the Ken Blanchard Companies approved the ethics of the study's design and procedures.

Informed consent was obtained from all individual participants included in the study using the text provided to participants in the electronic survey's beginning instructions, as shown verbatim in italics below. The following informed consent text was adapted from a version of survey instruction text approved by the IRB through the University of San Diego for a 2012 study with similar instructions. For this study, the adapted instruction text was approved by the Research Ethics Committee at the Ken Blanchard Companies. We did not provide participants with a printed paper version of an informed consent form, due to the electronic format of the survey. However, participants indicated their voluntary agreement to participate by reading the below instructions and clicking forward into the survey. In that text, we made clear the researcher's promise to keep data anonymous and to only report individual data in aggregate form, and that participants could quit at any time without penalty.

“*The purpose of this questionnaire is to assess the extent to which certain leader behaviors impact employee work intentions. You are being asked to participate in a survey that will take about 15–20 min to complete. Completion of this survey involves no foreseeable risks. Your participation is voluntary and you may stop at any time with no penalty. No one will see your individual responses other than the researchers. Any data will be reported on a group basis only. You give your consent to participate in the study by completing this survey. If you have any questions please contact xxxxxx@xxxx.com.”*

For background, individuals invited to the survey had previously opted-in to receive electronic survey invitations of this kind, as their emails were being housed by a consulting company's national listserv database. This listserv and process has been used by The Ken Blanchard Companies to conduct survey research for the last 15 years, particularly in the areas of engagement and work passion for companies all over the globe. Protecting survey respondent confidentiality and ensuring participant psychological well-being has been a necessary precondition required by participating companies and individuals.

## Author Contributions

TP led the data cleaning, data analysis, results write-up, and table/figure creation for both studies and is the corresponding author. DZ designed both studies, oversaw data collection, and wrote the literature review and discussion section. SF contributed to the literature review and discussion section, and all authors reviewed the piece prior to submission.

### Conflict of Interest Statement

DZ is affiliated to the Ken Blanchard Companies through an advisory role, but he is not an employee of Blanchard. SF is Principal of Out of the Box Learning, Inc. (i.e., self-employed, author). TP is Principal of Valencore, LLC (i.e., at the time of this study she was self-employed). DZ and SF have both been employed by the University of San Diego for 20 years as adjuncts in the Master's of Executive Leadership program, where they teach two courses. TP is currently employed by Boston University's School of Hospitality Administration as a full-time faculty member.
